# Role of Platelet-Derived Growth Factor (PDGF) in Asthma as an Immunoregulatory Factor Mediating Airway Remodeling and Possible Pharmacological Target

**DOI:** 10.3389/fphar.2020.00047

**Published:** 2020-02-14

**Authors:** Grzegorz Kardas, Agnieszka Daszyńska-Kardas, Mateusz Marynowski, Oliwia Brząkalska, Piotr Kuna, Michał Panek

**Affiliations:** Clinic of Internal Diseases, Asthma and Allergy, Medical University of Lodz, Łódź, Poland

**Keywords:** asthma, platelet-derived growth factor, PDGF = platelet-derived growth factor, airway remodeling in asthma, airway remodeling

## Abstract

Asthma is a chronic and heterogenic disease of the respiratory system, one of the most common lung diseases worldwide. The underlying pathologies, which are chronic inflammatory process and airway remodeling (AR), are mediated by numerous cells and cytokines. Particularly interesting in this field is the platelet-derived growth factor (PDGF), one of the members of the human growth factor family. In this article, the authors analyze the available data on the role of PDGF in asthma in experimental models and in human research. PDGF is expressed in airway by various cells contributing to asthma pathogenesis—mast cells, eosinophils, and airway epithelial cells. Research confirms the thesis that this factor is also secreted by these cells in the course of asthma. The main effects of PDGF on bronchi are the proliferation of airway smooth muscle (ASM) cells, migration of ASM cells into the epithelium and enhanced collagen synthesis by lung fibroblasts. The importance of AR in asthma is well recognized and new therapies should also aim to manage it, possibly targeting PDGFRs. Further studies on new and already existing drugs, mediating the PDGF signaling and related to asthma are necessary. Several promising drugs from the tyrosine kinase inhibitors group, including nilotinib, imatinib masitinib, and sunitinib, are currently being clinically tested and other molecules are likely to emerge in this field.

## Introduction

Asthma is one of the most common lung diseases with over 241 million prevalent cases reported worldwide in 2013 (Global Burden of Disease Study 2013 Collaborators GB of DS 2013, 2015). It is a chronic and heterogenic disease in which reversible obstruction of airway is observed. Two main underlying pathologies of the disease are the chronic inflammatory process and airway remodeling (AR). The two processes are mediated by numerous cells and cytokines in response to different environmental factors, particularly inhalable allergens. The chronic inflammation of airway epithelium plays a significant role in AR (HOLGATE ST, 2008). This pathology leads to changes in micro-vascularization, thickening of the airway walls and impaired air flow through the bronchi, and consequently, to ventilation impairment. However, it is not yet established whether the two pathologies are happening independently or whether one is triggering the other (Girodet et al., 2011).

Airway remodeling (AR) is a change in the composition, content, and distribution of cellular and molecular constituents of the airway wall (Bergeron and Boulet, 2006). In asthma, it involves many structural changes—epithelial damage, subepithelial fibrosis, angiogenesis, myofibroblasts, and myocytes hyperplasia and increased number of smooth muscle fibers in airway smooth muscle (ASM) cells that results in increased ASM mass. Currently, the possible role of epithelial mesenchymal transition (EMT) in this process is also strongly discussed. EMT is a process leading to transformation of epithelial cells into mesenchymal-like cells by loss of their epithelial functions and characteristics (Kalluri and Weinberg, 2009). Data regarding the presence and traits of EMT in AR in asthma are controversial and intensively investigated. Reviews of this subject do not yet answer the question whether EMT takes place in AR in asthma due to lack of *in vivo* evidence of this process. However, the authors suggest a need for further research in this area in order to extend the knowledge of the pathogenesis of asthma (Hackett, 2012; Bartis et al., 2014; Sohal et al., 2014).

As a result of these pathological changes, patients may experience an irreversible airway obstruction that does not respond to conventional asthma treatment. The process leads to other detrimental effects, such as decline in lung function, airway distensibility and bronchodilator response. AR contributes to the development and persistence of airway hyperresponsiveness and clinical symptoms (Pascual and Peters, 2005; James and Wenzel, 2007; Halwani et al., 2010; Boulet, 2018).

Growth factors—proteins regulating the divisions and differentiations of cells—involved in asthma include: Epidermal Growth Factor (EGF), Fibroblast Growth Factor (FGF), Transforming Growth Factors (TGFs), Vascular Endothelial Growth Factor (VEGF), Platelet Derived Growth Factor (PDGF)—described in more detail in this article—and other.

The activation of T-helper type 2 lymphocytes (Th2) by inhaled allergens has been traditionally considered as the primary cause of asthma. In response to stimulus (allergen, virus or oxidation) the epithelium cells secrete cytokines (e.g. TSLP, IL-25, IL-33) that trigger the underlying inflammation which is later moderated by allergen-specific IgE produced by B cells, mast cells, basophils, and eosinophils (Fahy, 2015). A significant role in the pathogenesis of asthma is also attributed to Group 2 innate lymphoid cells (ILC2), co-responsible with the Th2 for type 2 inflammation (Cosmi et al., 2017).

Defects in the airway epithelium barrier function are considered to facilitate penetration of environmental factors, such as inhaled allergens and pollutant particles, into the airway wall. Impaired repair of the epithelium in chronic asthma is also a factor that contributes to AR (Holgate, 2007).

PDGF is a member of the human growth factor family that regulates cell growth and division. It was originally discovered as a constituent of wholeblood serum, absent in cell-free plasma-derived serum (Kohler and Lipton, 1974; Ross et al., 1974; Westermark and Wasteson, 1976). Subsequently, this factor was isolated from human platelets (Heldin et al., 1979; Deuel et al., 1981; Antoniades et al., 1979). The primary source of this factor are α-granulates from activated platelets (Kaplan et al., 1979; Blair and Flaumenhaft, 2009). Many other cells, however, also express this factor, including fibroblasts, vascular smooth muscle and endothelial cells, macrophages, neurons and many other (Heldin and Westermark, 1999). The primary function of PDGF is the growth control of mesenchymal cells such as fibroblasts and smooth muscle cells (Kohler and Lipton, 1974; Ross et al., 1974).

Recently, research on PDGF provided novel information on its rolein AR in asthma (Hirota et al., 2011). As the factor is considered to strongly contribute to the AR process, it is frequently used as a positive control growth stimulus for airway smooth muscle (ASM) proliferation, particularly in search of new potential pharmacological candidates inhibiting the process of remodeling (Liang et al., 2017). In the further parts of this article, we discuss the role of PDGF in asthma pathophysiology, particularly in AR and highlight the possible trends in development of PDGF-oriented drugs.

### PDGF Structure and Its Receptor (PDGFR)

The PDGF family consists of four polypeptide chains encoded by four different genes. PDGFA, PDGFB, PDGFC, and PDGFD genes are located on chromosomes 7, 22, 4, and 11, respectively (Fredriksson et al., 2004). The first two members of PDGF family, PDGF-A and PDGF-B, were discovered in the 1970s, while the latter two, PDGF-C and PDGF-D were discovered in 2000 and 2001, respectively (Li et al., 2000; LaRochelle et al., 2001; Bergsten et al., 2001). These chains form five isoforms: PDGF-AA, PDGF-BB, PDGF-AB, PDGF-CC, and PDGF-DD.

All four PDGFs possess 18-22 amino acids long signals sequences with small homology between different types. The following parts, both in PDGF-A and PDGF-B, are the ~60 amino acids pro-peptide sequences which are intracellularly cleaved from the mature growth factors by furin or other proprotein convertases already before secretion (Heldin and Westermark, 1999; Fredriksson et al., 2004). On the other hand, PDGF-C and PDGF-D are proteolytically cleaved in the extracellular space (Reigstad et al., 2005). The growth factor core domain, which is in fact the same domain as in VEGF, is followed by a highly polar tail in PDGF-A and PDGF-B (Reigstad et al., 2005; Andrae et al., 2008; Chen et al., 2013). The receptor for PDGF, PDGFR, is classified as receptor tyrosine kinase (RTK). They are transmembrane proteins composed of five extracellular immunoglobulin-like domains and a split intracellular tyrosine kinase domain. The two PDGFR chain isoforms form dimers upon binding the PDGF ligand, leading to three possible dimer combinations: -αα, -ββ, and –αβ (Heldin and Lennartsson, 2013).

The protein thus acts *via* two receptors—PDGFRα, PDGFRβ. which are encoded on chromosomes 4 and 5, respectively. They belong to class III receptor tyrosine kinases (RTKs), which also includes macrophage colony-stimulating factor receptor (M-CSFR), Mast/stem cell growth factor receptor (SCFR, also known as C-KIT) and fms like tyrosine kinase 3 (FLT3) (Ségaliny et al., 2015). All of PDGF isoforms, except PDGF-DD, bind to PDGFRα, while PDGFRβ is activated by PDGF-BB and -DD. A heterodimeric form – PDGFRαβ - binds PDGF-AB, PDGF-BB and possibly PDGF-CC and PDGF-DD (Fredriksson et al., 2004; Andrae et al., 2008; Demoulin and Essaghir, 2014; Chen et al., 2015).

PDGFR may also bind vascular endothelial growth factor (VEGF), another growth factor closely related to PDGF, which also contributes in cell migration and proliferation (Ball et al., 2007). Recent findings also show that all PDGF dimers, except PDGF-DD, bind to vascular endothelial growth factor receptor 2 (VEGFR2). (Mamer et al., 2017) Structural similarities of PDGF and VEGF and their cross-family ligand-receptor interactions indicate their strong functional relationship.

PDGFR activation results in intracellular signaling pathways including Ras/Rac, MAPK, PI3K, STA Src and other, which subsequently promote cell proliferation and migration (Heldin and Westermark, 1999). The PDGFR pathway is discussed in more detail in the cited review (Demoulin and Essaghir, 2014).

### The Role of PDGF in Physiology

PDGF is strongly involved in embryogenesis by stimulation of cell proliferation and migration (Andrae et al., 2008; Demoulin and Essaghir, 2014). PDGFRα-dependent signaling controls gastrulation and the development of lungs, intestines, skin, testis, kidneys and other organs. The PDGFRβ signaling was observed in early hematopoiesis and blood vessel formation (Andrae et al., 2008). Unfortunately, the data on its physiological functions in adults is limited, mostly because of early lethality of mice with disruptions in PDGF genes (Demoulin and Essaghir, 2014). In adulthood, PDGF–PDGFR signaling is strictly controlled. The secretion of PDGFs by platelets and their effect on fibroblasts suggest their involvement in wound healing (Andrae et al., 2008). Except from wound repair, it is generally perceived abnormal, as it contributes in a number of proliferatory diseases—cancers, inflammatory diseases, pulmonary fibrosis, and restenosis, atherosclerosis. Its role has been recently intensely studied in chronic obstructive diseases, particularly in asthma and COPD.

PDGF is synthesized in bone marrow megakaryocytes, stored in α-granules and secreted by platelets upon activation, which triggers fibroblast activity. The protein is also released by various other cells in human body, including endothelial, epithelial, glial and inflammatory cells (Demoulin and Essaghir, 2014). PDGF-A and PDGF-B are cleaved and activated intracellularly, while the other two PDGFs are cleaved and activated extracellularly. Their synthesis is increased as a response to external stimulus such as exposure to hypoxia (Kourembanas et al., 1997), thrombin (Harlan et al., 1986) and various other growth factors and cytokines. PDGF-A expression is repressed e.g. by glucocorticoid treatment of smooth muscle cells (Nakano et al., 1993) and aging of human fibroblasts (Karlsson and Paulsson, 1994). PDGFs often act as paracrine factors; however, they may also act in autocrine pathways in tumors (Andrae et al., 2008).

### Pathophysiology of Airway Remodeling *via* PDGF

PDGF is expressed in airway by various cells contributing to asthma—mast cells, eosinophils, and airway epithelial cells. Mast cells secrete PDGF, along with other growth factors, including VEGF, bFGF, TGFβ, GM-CSK, and PAF (Zanini et al., 2010). Human eosinophils express PDGF-B, both in peripheral blood and in inflamed airway tissue (bronchial tissue and nasal polyps) (Ohno et al., 1995). The expression of PDGF has been proven both in mouse broncho-alveolar epithelial cells (spontaneously and in response to such factors as TNFα and TGFβ1) (Warshamana et al., 2001) and in human lung epithelial cells, where it is triggered by thrombin receptor PAR-1 (Shimizu et al., 2000). Other sources of PDGF have not been yet clearly demonstrated in asthma.

The first of the important effects of PDGF on AR is its impact on fibroblast proliferation. The effect of three PDGF isoforms (PDGF-AA, -BB, -AB) on human baseline airway fibroblasts was studied. Statistically significant increased procollagen I expression in cells from severe asthmatics was observed after PDGF-BB exposure, compared to fibroblasts from healthy controls. Also, significantly higher expression of PDGFRβ was observed in these cells. This study supports the thesis that PDGF-BB and PDGFRβ are presumably mostly involved in AR in asthma out of other isoforms of PDGF and PDGFR (Lewis et al., 2005). On the other hand, PDGF-AA has been observed as an autocrine factor mediating IL-13-induced proliferation of mouse, rat and human fibroblasts. The release of PDGF-AA, dependent on IL-13 exposure, was recognized to be mediated by STAT-6 signaling. Moreover, it was observed that IL-1β synergistically enhances this process by PDGFRα up-regulation, which suggests a synergic effect of these two interleukins in PDGF-AA/PDGFRα-dependent fibroblast proliferation (Ingram et al., 2003; JL et al., 2004).

α-2-macroblobulin (A2M) is one of the main factors contributing to PDGF regulation. A synergic effect of receptor-recognized A2M and PDGF on fibroblast proliferation was observed, possibly by an increase in local PDGF concentration at cell surface (Bonner et al., 1990). As the oxidative stress from inflammatory cells (eosinophils and neutrophils) impairs A2M, this may lead to enhanced PDGF signaling, which may be another way to explain the role of this factor in AR (Bonner, 1994).

The effect of PDGF in asthma on ASM is its next important role. Numerous studies have suggested that ASM migration towards airway epithelium, driven by inflammatory mediators such as PDGF, is a great factor contributing to AR (Hedges et al., 1999; Carlin et al., 2003; Gerthoffer, 2008; Suganuma et al., 2012). This change in smooth muscle cell localization results in their close proximity to epithelial cells (James et al., 2002), which may contribute to airway hyperresponsiveness.

*In vitro* studies strongly support the thesis that PDGF is a great mitogenic factor for human ASM cells. Hirst et al. studied the role of three PDGF isoforms – PDGF –AA, -AB and –BB on these cells. In absence of fetal calf serum (FCS), the PDGF-BB and PDGF-AB isoforms were potent mitogens, while PDGF-AA was weakly mitogenic. In presence of FCS, all of these isoforms stimulated ASM proliferation with similar efficiency. It was estimated in this study that ASM culture cells express 5 to 6 times more PDGFRβ than PDGFRα in response to stimulation with PDGF isoforms. In this study, PDGF-BB and its receptor—PDGFRβ—proved to be mostly involved in the ASM proliferation process compared to other two isoforms and PDGFRα (Hirst et al., 1996).

Hirota et al. performed studies in murine model of asthma to clarify the role of PDGF in ASM remodeling *in vivo*. Mice underwent treatment of adenovirus overexpression system technology (single intratracheal instillation of 5 x 10^8^ plaqueforming units of AdPDGF-BB) to selectively overexpress PDGF-BB in airway epithelium. Compared to control, those mice showed increased ASM area and ASM cell number, but not index of cell size. They also showed reduced expression of α-SMA and SM-MHC II gene transcripts. The airway responsiveness to nebulized methacholine was increased and the total lung compliance was decreased due to over-expression of PDGF-BB. The group also tested the bronchoalveolar levels of PDGF-BB after chronic allergen exposure (118 days intraperitoneal ovalbumin (OVA) exposure protocol). The protein level was significantly higher 24 h post 90 days OVA protocol allergen exposure, but it was not significantly different 4 weeks after the exposure was finished (Hirota et al., 2011).

The studies discussed above indicate the role of PDGF in two processes: increased synthesis of collagen by fibroblasts and significant impact on ASM—an increase in the area and cell number together with cell migration towards the epithelium. To prove the important role of this factor in asthma, it is necessary to confirm the differences in its expression in the course of the disease, and to verify whether it depends on the severity of the disease or the diagnosis itself. Many studies analyzed differences in PDGF expression in asthma, but their results are not unambiguous.

One of the first *in vivo* studies on PDGF expression pattern in humans with asthma and COPD, published in 1994, showed that PDGF(B) and PDGFRβ mRNA expression in lungs of patients with asthma did not statistically differ from healthy controls. However, these levels were significantly higher than in COPD patients (Aubert et al., 1994).

The levels of PDGF-AA and PDGF-BB in serum and induced sputum were analyzed by Zou et al. No significant differences in the expression levels of these factors were observed concerning the phenotype of asthma (eosinophilic and neutrofilic phenotypes) and the severity of asthma (mild/moderate/severe). Interestingly, the PDGF-AA levels appeared to be significantly higher in induced sputum than in serum, while PDGF-BB levels were significantly higher in serum than in induced sputum. In a follow-up of this study, which included 9 patients who received standardized treatment, consisting of short-acting β-agonists as required, inhaled corticosteroids plus long-acting β-agonists and leukotriene modifiers (no data on names of drugs used), no significant differences in the levels of the two PDGF isoforms neither in serum nor in induced sputum were observed (ZOU et al., 2014).

Another study showed no statistical differences in PDGF-BB levels in asthma vs. healthy control bronchialveolar lavage (BAL). However, the levels of PDGF were significantly higher in BAL fluid in eosinophilic asthma compared to non-eosinophilic asthma (Hosoki et al., 2015).

It was observed that PDGF-BB levels in exhaled breath concentrate positively correlate with Fractional exhaled nitric oxide (FeNO) levels in children with severe/refractory asthma (Brzozowska et al., 2015).

The aforementioned studies do not determine whether PDGF is in any way a systemic biomarker of asthma. It seems that it is a factor more involved in local (autocrine and paracrine) interactions than systemic ones.

Inhalable PM10 particles from urban air pollution have been demonstrated to enhance PDGFRα expression in lung myofibroblasts (Bonner et al., 1998). This study complements the complex mechanism of PDGF/PDGFR-dependent effects on airway in asthma.

**Figure 1:**
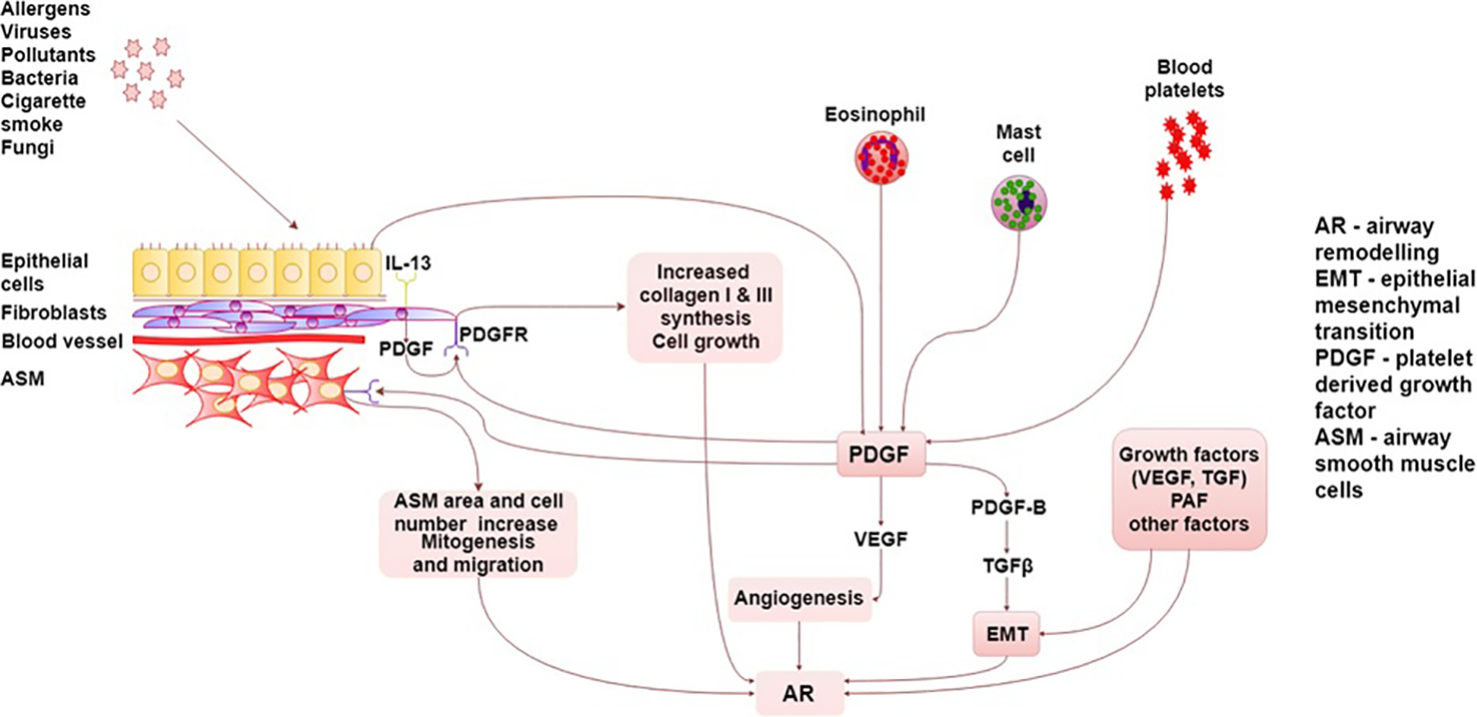
Summary of PDGF/PDGFR interactions in asthma. AR, airway remodeling; ASM, Airway smooth muscle; EMT, epithelial–mesenchymal transition; TGF, transforming growth factor; PAF, platelet activating factor; PDGF, platelet-derived growth factor, PDGFR, platelet-derived growth factor receptor; VEGF, vascular endothelial growth factor.

The summary of PDGF/PDGFR interactions in asthma are presented graphically in detail in **Figure 1**.

### PDGF and Its Inhibitors as a Possible Future Pharmacological Target

Due to its role in AR in asthma, PDGF and PDGFR inhibitors have become a potential pharmacological target. Several particles, being functionally tyrosine kinase inhibitors (TKI), which inhibit PDGFR and other members of this receptor group, were tested in asthma therapy.

Recent studies, concerning two structurally related TKI—imatinib and nilotinib, are worth mentioning. Nilotinib proved to suppress AR in murine asthma model. Both nilotinib and imatinib reduced the total cell number, macrophages, eosinophils, and neutrophils in BAL fluid with comparable efficacy. The two drugs also attenuated AR by inhibition of collagen deposition and smooth muscle layer thickening (Rhee et al., 2014; Kang et al., 2016). In a randomized, double-blind, placebo-controlled, phase 2 study (62 patients randomized, 50 finished the study), carried out by Cahill et al., 400 mg/day oral dose of imatinib decreased airway hyperresponsiveness, mast-cell counts, and tryptase release (Cahill et al., 2017). Until today, no data on phase 3 study of this drug is available.

Masitinib is another promising TKI targeting PDGFR. In a feline model of asthma, this drug reduced BAL eosinophilia and total protein, which indicated an improvement in airway inflammation and edema (Lee-Fowler et al., 2012). Phase 2, placebo-controlled study on masitinib in 44 patients with poor asthma control despite optimal treatment was reported in 2009. In this trial, a reduction of oral corticosteroids dosage was comparable in study and placebo groups; however, a significant reduction in Asthma Control Questionnaire score was observed (0.99 unit at week 16 in study group vs. 0.43 unit in the placebo group) (Humbert et al., 2009). Two phase 3 studies of masitinib among severe asthmatics began in June 2011 and December 2016. However, no results of these experiments have yet been reported (Masitinib in Treatment of Patients With Severe Persistent Asthma Treated With Oral Corticosteroids - Full Text View - ClinicalTrials.gov; Masitinib in the Treatment of Patients With Severe Uncontrolled Asthma and Elevated Eosinophil Levels - Full Text View - ClinicalTrials.gov.).

Another example is sunitinib—a multitaregting TKI inhibiting PDGFR, VEGFR, c-kit and other. The drug suppressed eosinophilic airway inflammation, AHR and airway remodeling in a murine asthma model, at least partially *via* PDGFR inhibition. It also reduced the total serum IgE and BALF Th2 cytokines (Huang et al., 2009). Lam et al. observed reduced airway hyperreactivity in house dust challenged mice, but with no reduction of mRNA levels of Th2 inflammation-associated genes (IL-13) and other mediators of airway hyperreactivity nor in the BAL inflammatory cell numbers (Lam et al., 2010). Although these results seemed promising, these are the only two available studies of sunitinib in asthma (Huang et al., 2009).

A different approach was taken by Ambhore et al. who studied *in vitro* the role of various estrogen receptor (ER) agonists and their effect on PDGF-induced ASM proliferation. It was demonstrated that ERβ activation significantly suppresses PDGF-stimulated human ASM cell proliferation and also arrests cell cycle progression in G0/G1 phase (Ambhore et al., 2018).

Numerous pharmacological candidates targeting PDGFR can attenuate ASM proliferation *in vitro*; however, there are no *in vivo* animal and human studies on their effectiveness. However, *in vitro* studies show promising results of Dimethylfumarate (DMF) (Seidel et al., 2010), baicalin (Yang et al., 2015), CC10 protein (Wei et al., 2013), iptakalim (Liu et al., 2015), S100A8 (Xu et al., 2016; Xu et al., 2017) in this area. Their effects are further discussed in **Table 1**.

**Table 1:** Summary of research on PDGF/PDGFR-related pharmaceutical targets.

Drug name	Drug type	Target	Model	Main effect	Source
Nilotinib	Tyrosine kinase inhibitor	PDGFR	Murine model of asthma remodelling (Ovalbumin sensitization)	Suppression of ASM fibrotic changes, inhibition of collagen deposition TGF-β1 in BAL reduction	(Kang et al., 2016)
				Reduced number of total cells, macrophages, eosinophils, and neutrophils in the BAL fluid AHR – decreased Peth value Decreased collagen (hydroxyproline) deposition Inhbition of fibroblast proliferation Inhibition of ASM area increase Decrease in expression of PDGFRβ mRNA	(Rhee et al., 2014)
Imatinib	Tyrosine kinase inhibitor	PDGFR; (also: ABL, c-kit)	Murine model of asthma remodelling (Ovalbumin sensitization)	Imatinib therapy in OVA-challenged mice significantly reduced the total number of cells, eosinophils and neutrophils in BAL fluid AHR – decreased Peth value Decreased collagen (hydroxyproline) deposition Reduced area of the ASM Layer TGF-β1 in BAL reduction SCF expression reduction	(Rhee et al., 2011)
			Randomized, double-blind, placebo-controlled, 24-week trial of imatinib in 62 patients with poorly controlled severe asthma	reduced airway hyperresponsiveness to a greater extent than did placebo increase in methacholine PC_20_ was more than 1 doubling dose Decrease in mast-cell counts, and tryptase release	(Cahill et al., 2017)
Masitinib	Tyrosine kinase inhibitor	PDGFR; (also: c-kit, Lyn, FGFR3)	Feline model of asthma (Bermuda grass allergen sensitization)	Reduced BAL eosinophilia and total protein	(Lee-Fowler et al., 2012)
			A 16-week randomized, dose-ranging (3, 4.5, and 6 mg/kg/day), placebo-controlled study in 44 patients	ACQ score improvement (reduction by 0.99 unit at week 16 vs 0.43 in the placebo arm)	(Humbert et al., 2009)
Sunitinib	Tyrosine kinase inhibitor	All PDGFRs; (also: all VEGFRs, c-kit, RET, CD114, CD135)	Murine model of asthma remodelling (Ovalbumin sensitization)	Supressed eosinophilic airway inflammation, AHR and airway remodeling in a murine chronic asthma model, at least partially *via* PDGFR inhibition. It also reduced the total serum IgE and BALF Th2 cytokines	(Huang et al., 2009)
			Murine model of asthma (House dust mite-induced allergic asthma)	Reduced airway hyperreactivity	(Lam et al., 2010)
WAY-200070; FERB-033;, DiarylPropio-Nitrile	ERβ agonists	ERβ	*In vitro –* human ASM cells (asthmatic and non-asthmatic_	Suppression of PDGF-stimulated ASM cell proliferation	(Ambhore et al., 2018)
Dimethylfumarate (DMF)	Increase in HO-1 expression	Supression of PDGF-BB-induced ASM proliferation	*In vitro –* human ASM cells	DMF down-regulates PDGF-BB induced proliferation of ASMC through a GSH and p38 MAPK dependent induction of HO-1	(Seidel et al., 2010)
Baicalin	Flavone glycoside	Unknown (Possibly suppressing the MAPK signaling pathway)	*In vitro –* rat ASM cells	Inhibition of PDGF-induced ASM cell proliferation, cell migration	(Yang et al., 2015)
CC10 (Clara cell 10 kDa protein)	Recombinant Protein	Unknown (Possibly downregulation of cyclin D1 expression)	*In vitro –* rat ASM cells	Inhibition of PDGF-BB-Induced ASMCs Proliferation and suppression of PDGF-BB-induced ASMCs migration	(Wei et al., 2013)
Iptakalim	lipophilic para-amino compound	ATP-sensitive potassium channel opener	*In vitro –* human ASM cells	Inhibition of PDGF-BB-induced human ASMCs proliferation and migration; Blockage of PDGF-BB-stimulated S phase entry of human ASMCs	(Liu et al., 2015)
S100A8	Recombinant protein	Unknown (Possibly mediation by RAGE membrane receptor, TLR4 and CD36)	*In vitro –* rat ASM cells	Inhibition of the PDGF-induced proliferation of ASM	(Xu et al., 2017)
				inhibits the PDGF-induced migration of ASM	(Xu et al., 2016)

Due to its involvement in numerous pathophysiological processes in the lungs, many other PDGF/PDGFR-directed therapies are being developed in other diseases such as: idiopathic PAH, lung cancer, lung fibrosis, IPF, Lymphangioleiomyomatosis and other (Kanaan and Strange, 2017). The use of multitarget TKI is already approved for treatment of lung cancer and pulmonary fibrosis. Thus, it is expected that drugs targeting PDGF will be used to treat other lung diseases, possibly including asthma.

## Conclusions

Platelet-derived growth factor is a well-recognized factor mediating the airway inflammation and remodeling in asthma. PDGF stimulates proliferation of ASM cells and migration of ASM cells into the epithelium and enhanced collagen synthesis by lung fibroblasts. Majority of studies of PDGF in asthma do not imply that this factor is a possible biomarker for the severity of the disease. No correlation of the levels of PDGF in serum and induced sputum and the severity were reported, with limited data on differences in PDGF expression dependent on asthma phenotype. However, this growth factor is considered one of primary growth factors contributing to AR. The importance of AR in asthma is well recognized and new therapies should also aim to manage it, possibly targeting PDGFRs. Further studies on new and already existing drugs, mediating the PDGF signaling and related to asthma are necessary. Several promising drugs from the TKI group, including nilotinib, imatinib masitinib, and sunitinib, are currently being clinically tested. Other molecules, currently in the basic research phase, are also likely to emerge in this research field.

## Author Contributions

GK and MP created the concept of the paper. GK and AD-K conducted the literature research and wrote the manuscript. PK, MP, MM, and OB revised the paper.

## Funding

GK is supported by grant no. 564/1-000-00/564-20-026 from Medical University of Lodz.

## Conflict of Interest

The authors declare that the research was conducted in the absence of any commercial or financial relationships that could be construed as a potential conflict of interest.
